# Triboelectric horology: escapement-inspired design strategy for prolonged energy harvesting under irregular mechanical inputs

**DOI:** 10.1038/s41378-026-01259-4

**Published:** 2026-04-13

**Authors:** Donghan Lee, Sanghu Ju, Dong Yong Park, Seokhoon Kwon, Yong Woo Jeong, Minsik Choi, Sangjun Lee, Yu-seop Kim, Sumin Cho, Dongik Kam, Zong-Hong Lin, Dongwhi Choi

**Affiliations:** 1 Department of Mechanical Engineering (Integrated Engineering Program), Kyung Hee University, 1732 Deogyeong-daero, Yongin, 17104 Gyeonggi Republic of Korea; 2 Advanced Mobility Components Group, Korea Institute of Industrial Technology, 320 Techno sunhwan-ro, Daegu, 42994 Republic of Korea; 3 Department of Biomedical Engineering, National Taiwan University, Taipei, 106319 Taiwan China

**Keywords:** Electrical and electronic engineering, Structural properties

## Abstract

The intermittent nature of ambient mechanical energy sources such as wind, vibration, and human motion poses a significant challenge for the stable operation of triboelectric nanogenerators (TENGs). To address this issue, this study presents a long-lasting operable triboelectric nanogenerator (LONG), a novel TENG system that utilizes an escapement-based mechanical regulation strategy to convert irregular and low-frequency mechanical inputs into stable and continuous electrical outputs. LONG integrates a rotational TENG system and an escapement mechanism to achieve a controlled and unidirectional rotational motion, thereby prolonging the energy-harvesting duration while minimizing the energy losses due to torque fluctuation. A freestanding-mode TENG, enhanced with electret materials fabricated via corona discharge are adopted to ensure efficient charge induction under low-torque conditions. Parametric studies reveal that key design variables, including spring tension, gear ratio, electret surface potential, and applied mass, significantly influence the output stability and voltage amplitude. The optimized LONG system demonstrated a peak voltage of 300 V, a current of 19 μA, and continuous operation exceeding 3 min from a single winding. Furthermore, its ability to power 125 LEDs, a thermo-hygrometer, and a dust collecting system validates its practical applicability in self-powered systems. Thus, this study introduces a robust design framework for mechanical regulation in TENGs, offering a new pathway for stable energy harvesting from irregular mechanical sources for wearable, environmental, and infrastructure-monitoring applications.

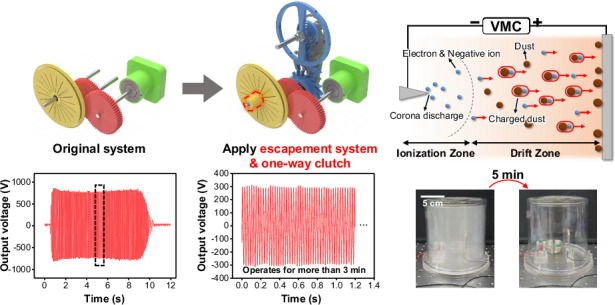

## Introduction

In recent years, concerns regarding the use of fossil fuels have intensified owing to the increasing severity of climate change, including global warming^1^. This growing urgency has driven a global shift toward cleaner and more sustainable alternatives, prompting extensive research on renewable energy technologies^2–10^. Among these, energy-harvesting systems have emerged as a promising solution, enabling the direct conversion of ambient energy into electricity without relying on a traditional power infrastructure^11–14^. In particular, mechanical energy-based energy-harvesting systems, such as triboelectric nanogenerators (TENGs), piezoelectric nanogenerators, and electromagnetic generators, have attracted growing interest because of their ability to utilize omnipresent mechanical stimuli in the environment^15–17^. Among these, TENGs, which generate electricity through contact electrification coupled with electrostatic induction, stand out because of their low cost, wide material versatility, lightweight construction, and structural simplicity^18–21^. These characteristics make TENGs ideal candidates for powering small decentralized devices, including wearable electronics, Internet of Things (IoT) sensors, and environmental monitoring systems. Moreover, their customizable structural design enables TENGs to be engineered for diverse applications at various scales and settings^22–25^. TENGs operate in several modes, including contact separation, lateral sliding, single electrode, and freestanding. Among these, the freestanding mode is particularly attractive because of its low torque requirement, absence of frictional resistance, and ease of continuous operation under low-force conditions. This mode enables a freely rotating triboelectric layer to interact with the stationary electrodes, facilitating efficient energy conversion even in low-energy environments.

However, ambient mechanical energy sources, such as wind, vibrations, and human motion, often exhibit irregularities in both frequency and intensity, presenting a critical challenge. This intrinsic intermittency leads to inconsistent and unstable electrical outputs when harvested directly, thereby limiting the long-term operational stability of TENG-based systems^26–28^. To address this issue, researchers have explored mechanical regulation strategies for converting irregular mechanical inputs into more stable outputs^29–33^. TENGs with these regulatory strategies have demonstrated sustainable electrical energy outputs from irregular mechanical input sources. Nonetheless, while these methods contribute to output stabilization, they often suffer from bulkiness, energy losses due to mechanical friction, and difficulty in harvesting low-frequency inputs. Therefore, compact and efficient regulation strategies that can effectively store and release mechanical energy in a controlled and periodic manner are required, particularly under infrequent or low-frequency input conditions.

This study proposes a long-lasting operable triboelectric nanogenerator (LONG), a novel TENG system designed to convert irregular and infrequent mechanical inputs into sustained and stable electrical outputs. The core innovation of LONG lies in the integration of an escapement mechanism, which is widely utilized in horology and precision mechanics. An escapement mechanism, which is widely used in timekeeping devices and precision mechanical systems, functions by regularly locking and releasing a rotating component, thereby preventing uncontrolled motion while ensuring a steady and regulated transfer of mechanical energy for consistent power generation^34,35^. This repetitive locking and releasing process ensures the generation of a consistent mechanical output from the stored input energy, regardless of the magnitude or frequency of the input energy. Consequently, this controlled release converts irregular mechanical excitation into consistent rotational motion, which in turn enables continuous energy generation by the TENG.

However, the regulation mechanism of the escapement system generates an intermittent rotational output owing to its cyclic locking and releasing motions, which can impede smooth operation of the rotor and result in unintended energy losses. To mitigate this issue, a one-way clutch bearing was incorporated at the output of the escapement, allowing for the smooth transmission of torque in a single direction while minimizing the energy loss due to backlash or reverse drag. This design enabled the LONG to effectively convert non-uniform mechanical input into regulated, unidirectional rotational motion. Furthermore, by integrating this mechanical regulation strategy with a freestanding-mode TENG enhanced by corona-discharged electrets, the system achieves efficient activation under low mechanical input while maintaining a high electrostatic output. Additionally, through a comprehensive parametric analysis and optimization of the escapement mechanism configuration, the study demonstrates the method by which the proposed design maximizes energy conversion efficiency while minimizing output fluctuation and energy dissipation. Consequently, the proposed LONG can serve as an alternative power source for applications requiring long-term stable power output, thereby offering an essential design strategy for high-quality mechanical energy-harvesting systems. Furthermore, the practical applicability of LONG as a self-powered system is validated through a self-powered air quality improvement application that successfully drives a dust collection system utilizing high voltage.

In summary, this study introduces an innovative mechanical design strategy that effectively overcomes the challenges of output instability and short operational duration by enabling the consistent harvesting of irregular low-frequency mechanical energy. The proposed strategy not only enhances the reliability and efficiency of TENG-based systems but also broadens their practical applicability to areas such as infrastructure sensing, and off-grid environmental quality control systems.

## Results and discussion

### Operation principles of LONG

**Fig. 1:**
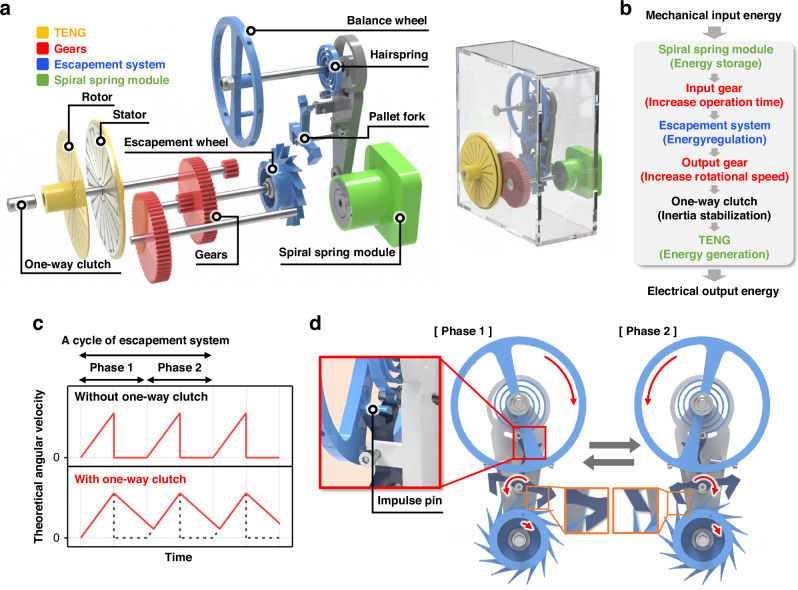
**Configuration and mechanism of LONG. a** Schematic representations of the composed and decomposed configurations. **b** Energy conversion process from mechanical input to electrical output. **c** Theoretical angular velocity of the output gear. **d** Schematic of the detailed operation mechanism of the escapement system

Prior to describing the configuration of LONG, a detailed explanation of the escapement system is provided to facilitate a more clear understanding of its operating principle. An escapement system is a mechanical device that converts potential and elastic energy into kinetic energy at a consistent speed. Because of its intrinsic complexity and precision, a carefully engineered structure is required. Generally, an escapement system comprises three key components: (1) the escapement wheel, which converts the stored input energy to regular rotational energy; (2) the pallet fork, which controls the rotation speed of the escapement wheel while transferring the input energy to the balance wheel; and (3) the balance wheel and hairspring, which regulate the periodic motion of the output rotation. Figure 1a presents a schematic of the structural configuration of LONG, which incorporates the escapement system. The overall configuration of LONG can be categorized into four functional parts: TENG, gears, the escapement system, and the spiral spring module. Spiral springs are typically used to store input energy. In this study, the spiral spring module stores mechanical energy in the form of elastic energy by manually pulling a wire. Therefore, a full cycle is defined as the process of fully utilizing the stored energy after a single winding allowing for quantitative comparison under various conditions. The role of each component is further described in Fig. 1b, which depicts the energy conversion process. Specifically, the mechanical input energy is initially stored as elastic energy in the spiral spring module and is then transmitted to the escapement system via the input gear. Here, since the rotational speed of the escapement wheel is determined by the mechanical structure of the balance wheel and hairspring, the input gear serves to reduce torque and extend the operation time. Once transferred to the escapement system, the mechanical energy is regulated into a consistent torque and angular velocity. Subsequently, the rotational speed of the regulated rotational motion increases via the output gear. However, as illustrated in Fig. 1c, the theoretical angular velocity of this rotational motion follows a cyclic pattern of acceleration and cessation owing to the locking and releasing mechanisms of the escapement system. This discontinuous motion results in a significant torque loss. To mitigate this energy loss, a one-way clutch is integrated into the system. This component ensures that the rotational inertia continues to drive the shaft even during intermittent stops of the escapement wheel, effectively enhancing the energy utilization efficiency. Finally, the rotational energy is converted into electrical energy using the TENG. Through this sequential energy-transformation process, the mechanical input energy is efficiently converted into a prolonged and regulated electrical output.

The fundamental working mechanism of the escapement system is illustrated in Fig. 1c, d. As shown in Fig. 1c, a complete cycle of the escapement system consists of two consecutive angular velocity peaks, each corresponding to a distinct phase. As illustrated in Fig. 1d, the operation of the escapement system is divided into two distinct phases characterized by alternating interactions among its key components. The balance wheel, positioned at the core of this mechanism, oscillates and plays a pivotal role in motion regulation. Attached to the balance wheel is an impulse pin, as shown in the enlarged schematic, which periodically engages the pallet fork to deliver mechanical impulses. During Phases 1 and 2, the oscillatory motion of the balance wheel causes the impulse pin to intermittently interact with the pallet fork, triggering it to rotate in alternating directions. At the beginning of the pallet fork rotation, a portion of the stored torque from the escapement wheel is transmitted to the pallet fork. This torque is then relayed through the impulse pin, thereby restoring and sustaining the rotational velocity of the balance wheel. Once the pallet fork completes its motion, it locks the tooth of the escapement wheel via its locking face, and momentarily halting its rotation. Through this cyclic locking and release process, each oscillation of the balance wheel advances the escapement wheel in a fixed angular increment corresponding to the spacing between successive teeth. Simultaneously, the hairspring, which stores elastic potential energy as it coils and uncoils, reverses the direction of the balance wheel’s rotation, initiating the next cycle. This coordinated interaction ensures a continuous and regulated release of mechanical energy, enabling stable and long-term rotational motion, which is essential for sustained energy harvesting. Electrical output generation.

After refinement through a one-way clutch, mechanical energy is transferred to rotate the rotor of the TENG, thereby producing an electrical output. In the LONG, a torque-reducing design is implemented to enhance the duration of electrical output from the limited mechanical input. Accordingly, among various operational modes of TENGs, the freestanding mode is adopted because of its relatively low torque requirement and ease of activation^22,36^. The freestanding mode is designed to minimize physical friction, thereby facilitating smooth rotor motion. However, this characteristic often results in insufficient triboelectric charge generation owing to the reduced contact-separation cycles. To compensate for this deficiency, electrets are introduced. An electret is a dielectric material with high electron affinity that can retain a quasi-permanent surface electrostatic field by storing a large quantity of surface charges^37–40^. In this study, an electret is fabricated using the corona-discharge method. This technique uses the corona discharge induced by a high-voltage supply to implant charges onto the surface of a dielectric film. This method enables precise control of the amount of surface charge and ensures long-term charge retention.

**Fig. 2:**
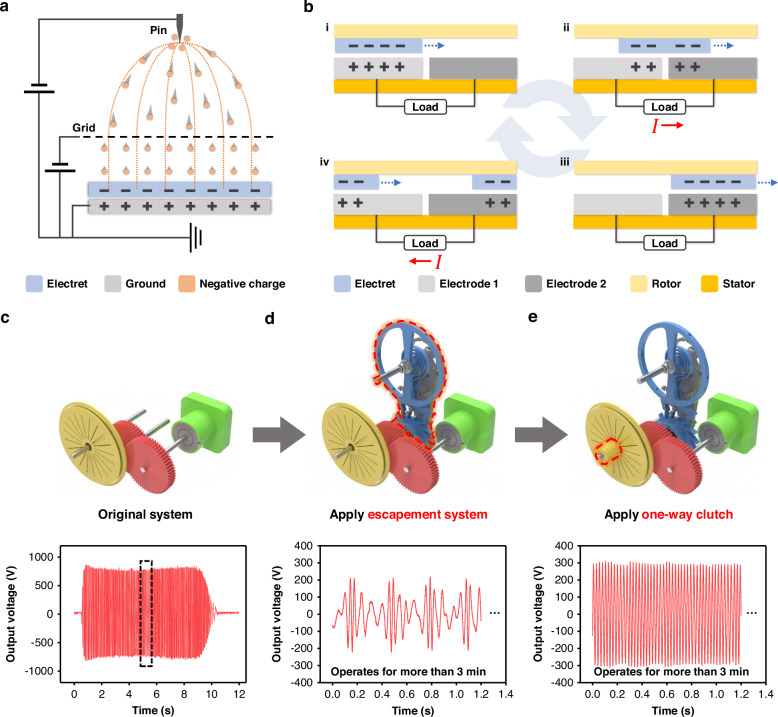
**Working principle of LONG for electrical output generation. a** Schematic of the corona discharge process used to fabricate electrets. **b** Schematic of the mechanism for electrical output generation via TENG. Electrical output depending on system configuration: **c** Original system, **d** With escapement system, **e** With one-way clutch

Figure 2a illustrates a schematic of the electret fabrication process via corona discharge. Under the influence of a high-voltage power supply, the tip of the pin electrode emits a corona discharge that ionizes the surrounding air, generating a large number of negative charges. These charges are attracted to the grounded electrode owing to the electrostatic field. As the densely accumulated charges near the tip of the pin pass through the grid electrode, they spread uniformly and are inserted into the surface of the dielectric film placed on the grounded electrode. The resulting electret exhibits a high surface potential, effectively inducing electrostatic charges in the electrodes on the stator during rotor rotation. Figure 2b presents a sequence of schematics detailing the mechanism of electrical output generation during the TENG operation. As shown in Fig. S1, the electrets are alternately arranged in a radial pattern on the rotor surface, whereas a pair of electrodes are interdigitally patterned on the stator. Initially, because of the negative charges embedded in the electret, positive charges are electrostatically induced in electrode 1 (Fig. 2b-i). When the rotor turns and the electret moves toward electrode 2, positive charges are transferred through the external load from electrodes 1 and 2 (Fig. 2b-ii). Once the electret fully overlaps with electrode 2, the maximum amount of positive charge is induced (Fig. 2b-iii). As the rotation continues and the electret returns toward electrode 1, the charges shift back through the load to electrode 1 (Fig. 2b-iv). This cyclical charge transfer continues as the rotor rotates, resulting in alternating current (AC) output from the TENG.

Figure 2c–e illustrate the effects of the key components of the LONG on electrical output from identical mechanical input energies along with the resulting output signals. The other structural parameters follow the optimal configurations adopted for the experimental studies discussed in subsequent sections. As shown in Fig. 2c, the original system, consisting of only the spiral spring module, gears, and TENG, generates an output voltage of approximately 700 V within 10 s during the full cycle of the spring. While this output is characterized by a high frequency and high voltage, as shown in the enlarged graph in Fig. S2, the duration is extremely short, indicating a transient operation with limited energy utilization. When the escapement system is incorporated, as shown in Fig. 2d, the output is regulated into a periodic sinusoidal waveform, enabling continuous operation for more than 3 min. However, because of the cyclic acceleration and cessation of the escapement mechanism previously explained in Fig. 1c, significant torque loss occurs during rotation. Finally, as shown in Fig. 2e, the integration of a one-way clutch between the rotor and shaft mitigates the torque loss and enables the generation of a more stable and continuous electrical output with a higher voltage amplitude. These findings experimentally demonstrate the capability of LONG to convert a single instance of mechanical input energy into a prolonged and stable electrical output. This validates the novelty of the proposed design and confirms its potential for long-term energy harvesting applications.

### Parametric study

**Fig. 3:**
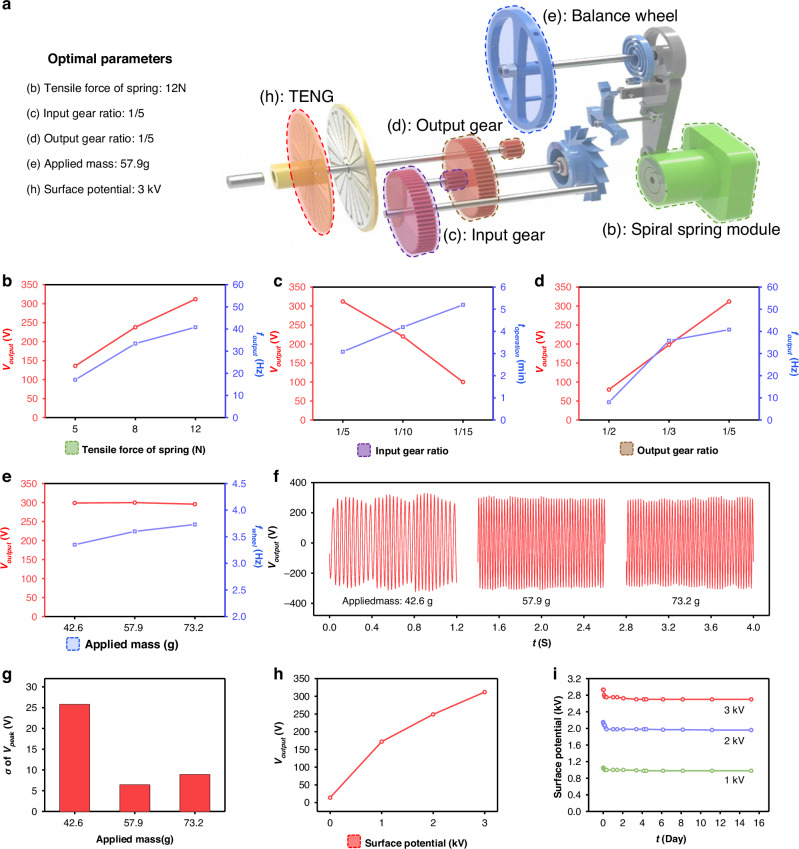
**Parametric studies of LONG**. **a** Schematic of the decomposed configuration and corresponding optimal parameters. **b** Output voltage and frequency as tensile force of spring. **c** Output voltage and operation time as input gear ratio. **d** Output voltage and frequency as output gear ratio. **e** Output voltage and escapement wheel frequency as applied mass. **f** Output voltage waveform under varying applied mass conditions. **g** Standard deviation of the peak voltage as applied mass. **h** Output voltage as surface potential. **i** Surface potential decay of electrets

Figure 3 presents a comprehensive parametric study conducted to identify and optimize the design parameters of the proposed LONG. The key parameters that potentially influence the electrical output of LONG include the tensile force of the spiral spring, input gear ratio, output gear ratio, mass applied to the escapement wheel, and surface potential of the electret. Each of these variables independently affects output performance. Therefore, rather than analyzing all the parameters simultaneously, a sequential analysis is performed by varying each parameter individually based on the optimal configuration. Figure 3a shows the optimal values obtained from the experiments for each parameter while mapping these values to the corresponding parts in the exploded view of the LONG. Alphabetical indicators (b)–(e) and (h) correspond to Fig. 3b–e, h, respectively. The notations used in the analysis are as follows: *V*_output_ (output peak voltage), *f*_output_ (output signal frequency), *t*_operation_ (operation time), *f*_wheel_ (escapement wheel frequency), and *t* (time).

Figure 3b shows the variations in *V*_output_ and *f*_output_ as functions of the tensile force of the spring. Because the tensile force represents the magnitude of the mechanical input, the results demonstrate an approximately proportional relationship between the input force and the electrical output. Therefore, a spiral spring with the maximum force of 12 N is identified as the most favorable choice for achieving a high output. Figure 3c illustrates the effects of the variations in the input gear ratio on *V*_output_ and *t*_operation_. Gear ratio is defined as the number of teeth on the driven gear relative to that on the driving gear. As the gear ratio decreases, a reduction in torque and an increase in rotation are induced. However, because the rotational speed of the escapement wheel is governed by the mechanical structure of the balance wheel and hairspring, the input gear cannot regulate the rotational speed of the output gear coupled to the escapement wheel. The results indicate that lower input gear ratios lead to a reduced torque transferred to the rotor, causing reduced *V*_output_ but extended *t*_operation_, suggesting that the input gear ratio plays a critical role in determining the duration of the energy output. Therefore, depending on the requirements, a higher input gear ratio can be chosen for a high-voltage output, whereas a lower input gear ratio is preferable for an extended operation time. In this study, an input gear ratio of 1:5 is selected to satisfy the high-voltage requirements for subsequent applications. As previously described, the rotational speed of the output gear coupled with the escapement wheel is determined by the components of the escapement system. Therefore, the output gear ratio, shown in Fig. 3d, governs the amplification of the rotation of the rotor after the escapement system. Similar to the input gear ratio, a lower output gear ratio increases rotation while reducing torque. The results reveal that reducing the gear ratio from 1:2 to 1:5 results in a consistent increase in *V*_output_. However, the rate of increase in *f*_output_ decreases significantly when the ratio changes from 1:3 to 1:5, implying that the reduced torque is insufficient to overcome the rotational resistance of the rotor. Thus, while a lower output gear ratio enhances the output voltage, it may compromise *V*_output_ and *f*_output_ owing to inadequate torque at lower gear ratios. Based on these findings, an output gear ratio of 1:5 is adopted to strike a balance between voltage amplitude and rotational stability. Figure 3e, f show the effects of applied mass on the escapement wheel. The attached mass increases the rotational moment of inertia, thereby requiring more force to initiate rotation, but contributing to a more stable and consistent rotational behavior. Three masses (42.6, 57.9, and 73.2 g) are tested. Although *V*_output_ remains relatively constant with increasing mass, *f*_wheel_ increases. This trend is attributed to the decrease in the angular displacement per torque impulse with increasing mass. As shown in Fig. 3f, *f*_wheel_ significantly affects the quality of the output signal. For 42.6 g, the frequency of the torque impulses transferred to the rotor is insufficient, resulting in large fluctuations in *V*_output_, even in the presence of the one-way clutch. The standard deviation of the peak voltage (σ of *V*_peak_), shown in Fig. 3g, is also significantly high at an applied mass of 42.6 g. These fluctuations can compromise the power supply stability for sensitive applications. Additional analysis is conducted to quantitatively assess this variability. Figure S3 shows the upper envelope of *V*_output_ for each applied mass. Although the average upper envelope of *V*_output_ for the three cases (290, 295, and 289 V) are similar, the standard deviations are 25.8, 6.44, and 9.82, respectively, confirming that the applied mass significantly influenced the output stability. This result suggests that securing a sufficiently high *f*_wheel_ is essential for maintaining consistent torque delivery and preventing rotor deceleration. However, an excessive mass can lead to higher energy consumption to drive the escapement wheel, resulting in reduced system efficiency. Therefore, 57.9 g is selected as the optimal mass, offering a trade-off between rotational stability and energy efficiency. Figure 3h shows the effect of the surface potential of electrets on *V*_output_. As the surface potential directly influences the electrostatic induction in the TENG, a higher potential leads to an increased electrical output. In addition, stability is as critical as the magnitude of the surface potential. Since low stability of surface potential can lead to a degradation in system output, it is a factor that must be evaluated. Figure 3i shows the surface potential decay over 15 days depending on the initial surface potential of the electret. Through the optimized electret fabrication setup and material utilization, the electrets exhibit excellent stability. Although a slight decrease in surface potential occurs immediately after fabrication, it stabilizes at approximately 90% of the initial value after about 6 h. While a higher surface potential leads to a larger initial decay, the fact that it stabilizes at a higher potential supports the justification for using electrets with high surface potential. Accordingly, the maximum surface potential of 3 kV, as determined by the corona-discharge fabrication method, is employed in the LONG. Given the intricate and sensitive nature of the escapement mechanism, rigorous parametric evaluation is essential. Each parameter plays a distinct role in determining the output quality, and therefore, they must be fine-tuned according to the specific operational requirements of the LONG. These findings not only validate the feasibility of integrating escapement mechanisms into TENGs for energy harvesting, but also provide valuable design insights for future studies employing similar regulation strategies.

### Electrical outputs

**Fig. 4:**
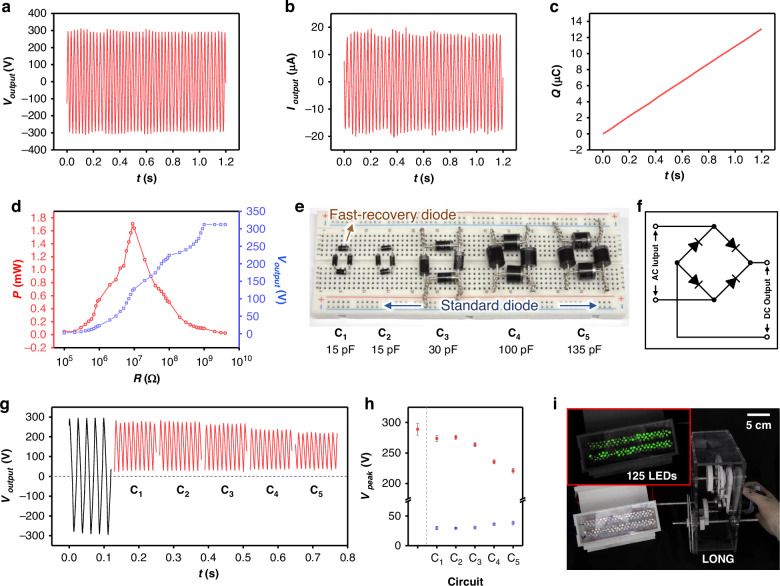
**Electrical outputs of LONG**. **a** Output voltage, **b** output current, and **c** transferred charge of LONG with its optimal configuration. **d** Output voltage and the corresponding power as a function of external resistance. **e** Photograph of electrical rectification circuit with different diodes. **f** Circuit diagram of a bridge circuit. **g** Raw and rectified output voltages for different circuits. **h** Maximum and minimum peak voltages for different circuits. **i** Photograph of the application activating the LED illumination

The fabricated LONG generates a high voltage of approximately 300 V and an output current (*I*_output_) of 19 μA, as shown in Fig. 4a, b. Because the output of the TENG appears as a high-frequency sinusoidal waveform, quantifying the transferred charge (*Q*) is essential. The rectified output yields a transferred charge of approximately 13 μC over 1.2 s, as illustrated in Fig. 4c. Figure 4d presents the variation in *V*_output_ and the corresponding power (*P*) as a function of the external resistance. As the resistance increases, *V*_output_ increases from 0 V and saturates at around 300 V near 1 GΩ. Accordingly, *P* increases with resistance, peaking at approximately 1.7 mW around 10 MΩ and subsequently decreasing. The power was calculated using the following equation:

1 $$P\,=\,{V}^{2}/R$$

This result suggests that the internal impedance of the LONG is approximately 10 MΩ, and the maximum power transfer occurs under this condition. This finding is significant, because it identifies the optimal load conditions for practical power delivery in external circuits.

For practical applications, the electrical output from LONG must be converted to a direct current output via rectification. Generally, diodes are used for the electrical rectification of energy harvesting system outputs. However, for TENGs characterized by very high voltages, selecting appropriate diodes for the rectification circuit is crucial. This is because the dynamic characteristics of the diode can affect the rectified TENG output. First, the reverse recovery characteristics of silicon-based diodes induce transient current conduction in the reverse direction during polarity switching, resulting in incomplete charge transfer and clipping of the output peak^41,42^. Furthermore, the intrinsic junction capacitance and non-negligible switching delay of the diodes act as low-pass filters, particularly under high ${dV}/{dt}$ conditions typical of high-voltage signals, suppressing sharp voltage transitions and attenuating the peak amplitude^41^. These effects are particularly pronounced in high-voltage bridge rectification systems, wherein the steep voltage slope and high current contribute to charge storage and recombination at the PN junction^42^. Moreover, owing to extremely low current levels, the diode may not operate within its ideal conduction regime. Under such conditions, the forward threshold voltage can increase slightly, and the device may exhibit highly nonlinear *I–V* characteristics, leading to reduced effective conduction and peak suppression^43^. Despite such output distortion caused by the dynamic characteristics of diodes, many TENG studies often construct rectification circuits simply using diodes with high rated voltages. Therefore, this study compares the influence of diode dynamic characteristics through rectification circuits (C_1_–C_5_) composed of different diodes, as shown in Fig. 4e. All circuits are fabricated as bridge circuits shown in Fig. 4f, using four diodes each. All circuits have a rated voltage of 1 kV. Additionally, C_1_ consists of fast recovery diodes with very short reverse recovery times, while C_2_–C_5_ consist of standard diodes. The individual diodes used in C_1_ to C_5_ have capacitances of 15, 15, 30, 100, and 135 pF, respectively. Figure 4g, h show the raw data of the rectified voltage and the maximum and minimum peak voltages (*V*_peak_), respectively, according to the circuit. Consequently, as the diode capacitance increases, the maximum *V*_peak_ decreases and the minimum *V*_peak_ increases, resulting in a flattening of the rectified output signal. Also, at the same diode capacitance, the difference in reverse recovery time does not have a significant effect. This is likely because commercial fast recovery diodes are designed for very high frequencies of tens of kHz or more, so they do not make a significant difference in cases with relatively low output frequencies like LONG. Thus, even if the diode has a sufficient rated voltage, using a diode with high capacitance may result in unintended output waveforms. In particular, since TENGs can be usefully utilized in high-voltage applications, a decrease in maximum voltage can reduce the practical utility of the TENG. In this study, C_1_, which maintains a high peak voltage, was finally adopted as the electrical rectification circuit, and as a result, 125 series-connected LEDs are effectively lit, as shown in Fig. 4i and Supporting Video 1.

### Practical applications

**Fig. 5:**
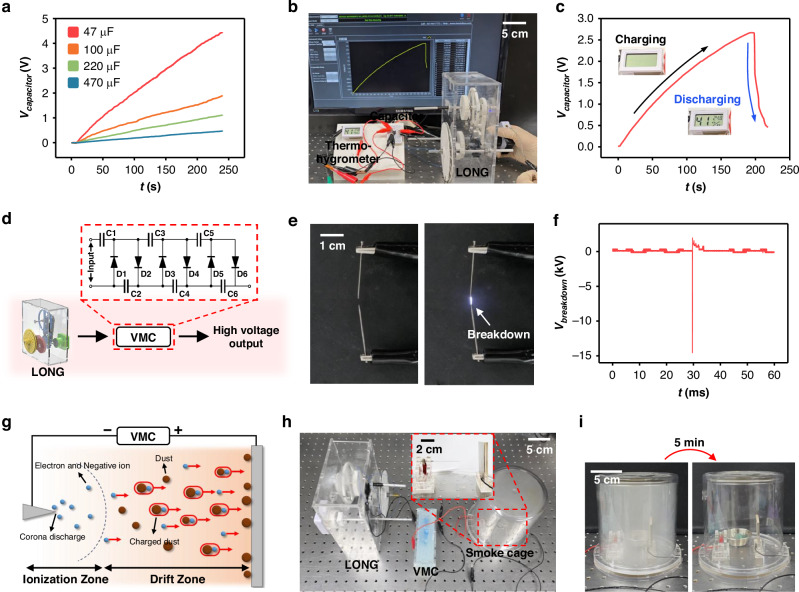
**Applications of LONG**. **a** Charging curve of capacitors under continuous rectified output. **b** Photograph of the experimental setup for operating the thermo-hygrometer, and **c** graph illustrating the voltage variation of capacitor of 47 μF during operation. **d** Schematic diagram of the process to boost the voltage using voltage multiplier circuit. **e** Photograph and **f** breakdown voltage of the spark generated between the needles. **g** Schematic diagram illustrating the working principle of the corona-discharge-based dust collection system. **h** Photograph of the experimental setup for the dust collection system. **i** Photographs of the smoke cage filled with smoke before and after operating the dust collection system

Figure 5 demonstrate the practical applicability of LONG in various scenarios. First, Fig. 5a–c introduce applications to demonstrate the feasibility of driving commercial small electronic devices. To evaluate the capability of the proposed LONG in supplying energy to low-voltage electronic devices and sensors, its ability to charge capacitive storage elements is assessed. Figure 5a shows the capacitor voltages (*V*_capacitor_) over time as the rectified output continuously charges them. During a 240 s operation, the LONG charges 47, 100, 200, and 470 μF capacitors to 4.42, 1.90, 1.11, and 0.46 V, respectively, demonstrating its potential for practical short-term power delivery. Figure 5b, c illustrate the activation of a small electronic device using the stored energy. Figure 5b shows the complete setup, in which a 47 μF capacitor is charged by the LONG and then used to power a digital thermo-hygrometer. By winding the spiral spring once, the capacitor is charged to approximately 2.7 V and is then used to operate the device. As shown in Supporting Video 2 and Fig. 5c, the charged capacitor activates the thermo-hygrometer for approximately 5 s after the circuit is connected.

Figure 5d–i show applications utilizing the high-voltage output of LONG. TENGs possess the intrinsic characteristic of having high voltages at the level of hundreds to thousands of volts. This characteristic enables self-powered operation of applications requiring high-voltage inputs. In this study, the high practicality of LONG is experimentally demonstrated through dust collection utilizing corona discharge based on high-voltage input. First, since corona discharge requires a voltage of several kV, it is necessary to further increase the output voltage of LONG. Therefore, a voltage multiplier circuit (VMC) is utilized to boost the voltage, as shown in Fig. 5d. The VMC used in this study consists of six capacitors and diodes each, and theoretically can accumulate voltage up to six times the input voltage^44^. The voltage accumulated through the VMC generates a spark with a breakdown voltage (*V*_breakdown_) of approximately 15 kV when two needles are facing each other at a certain distance, as shown in Fig. 5e, f. This indicates that the output of LONG can be accumulated to a voltage magnitude capable of corona discharge using a VMC. Figure 5g shows a schematic introducing the principle of dust collection technology utilizing corona discharge. The negative and positive poles of the VMC output are connected to the needle electrode and the plate electrode, respectively. When a voltage exceeding the threshold voltage for corona discharge accumulates in the VMC, corona discharge occurs at the tip of the needle, generating a large amount of electrons and negative ions within the ionization zone. These electrons and negative ions attach to surrounding dust particles, and the charged dust particles drift toward the plate electrode due to the electric field between the two electrodes. The detailed setup of the dust collection system using LONG is shown in Fig. 5h. The output of LONG is stored in the VMC, and the output of the VMC is connected to the needle electrode and plate electrode inside the smoke cage, respectively. The tip of the needle electrode and the plate electrode maintain a distance of 3 cm. Consequently, as shown in Fig. 5i, it has been experimentally demonstrated that most of the smoke particles filling the smoke cage are collected during the 5 min operation of LONG. This experimental demonstration implies that LONG possesses unique practicality compared to traditional electromagnetic generators.

These application demonstrations clearly validate the practical utility of LONG by successfully driving a thermo-hygrometer and a dust collection system. This study emphasizes the development of a novel escapement-based mechanical regulation strategy for TENGs, focusing on structural simplicity and ease of parametric optimization rather than a wide variety of applications. Future studies will explore system miniaturization and circuit optimization to further enhance the spatial efficiency and applicability of LONG. In conclusion, the mechanism proposed in this study provides a promising approach for converting irregular or single-shot mechanical inputs into stable electrical outputs, offering a meaningful solution to the limitations of existing energy harvesting technologies.

## Materials and methods

### Fabrication of the LONG

The overall dimensions of the LONG, which integrates the TENG, gears, escapement system, and spiral spring module, are 160 mm × 190 mm × 147 mm. The TENG, gears, and escapement system were fabricated using a 3D printer (3DF-210F, CUBICON, Republic of Korea) with polylactic acid (PLA) filament. The rotor of the TENG features ten electrets alternately attached on its surface using conductive double-sided tape. Correspondingly, the stator includes a pair of radial electrodes, each consisting of ten teeth, affixed to its surface. The diameters of both the rotor and the stator are 94 mm. The effective area of each electret is 200 mm^2^. A thrust bearing was used between the rotor and stator to allow the rotor to rotate while maintaining a constant distance from the stator. The spiral spring module, which stores elastic energy through mechanical winding, was adopted from a commercially available product. The housing supporting the entire system was fabricated from 5 mm-thick acrylic plates. All rotating shafts have a diameter of 6 mm and are combined with bearings and one-way clutches to ensure unidirectional rotational transfer.

### Fabrication of electrets

As illustrated in Fig. 2a, the electrets were fabricated by injecting negative charges into the surface of a 125 μm-thick fluorinated ethylene propylene (FEP) film via a corona discharge process. The FEP film was positioned on a grounded electrode during charging. The grid electrode consisted of an aluminum wire mesh with a 5 mm pitch. The distances from the ground electrode to the grid and pin electrodes were 1 cm and 8 cm, respectively. To fabricate electrets with surface potentials of 1, 2, and 3 kV, high-voltage supplies (HV30N, Nano NC, South Korea) were used to apply voltages of 1, 2, and 3 kV to the grid electrode, respectively, while maintaining the pin electrode at 14–15 kV for 2 min.

### Measurement of electrical outputs

The output voltage was measured using an oscilloscope (DS1074Z, Rigol, Beaverton, USA) with an input impedance of 1 MΩ, along with a high-voltage probe (DP-50, Pintek, New Taipei City, Taiwan, China) with an input impedance of 15 MΩ. Output current measurements were conducted using a low-noise current preamplifier (SR570, Stanford Research Systems, Sunnyvale, USA) in combination with the oscilloscope. During the capacitor charging experiments, the voltage across the capacitor was measured using an electrometer (Keithley 6514, Tektronix Co., USA). All measurements were performed at room temperature under approximately 40% relative humidity.

### Preparation of electrical rectification circuits

The rectification circuits were each fabricated as a bridge circuit using four commercial diodes. UF4007, 1N4007, 1N5408, 6A10, and 10A10 diodes were used for C_1_–C_5_, respectively. The rated voltage of each diode is 1 kV, and the diode capacitances are 15, 15, 30, 100, and 135 pF, respectively. The UF4007 is a fast recovery diode and was used to compare the effect of reverse recovery time on the output.

## Conclusion

This study introduces LONG, a triboelectric energy-harvesting system that leverages an escapement-based mechanical regulation strategy to overcome the fundamental limitations of conventional TENGs subjected to irregular or low-frequency mechanical inputs. By integrating a spiral spring for energy storage, a precision escapement mechanism for motion regulation, and a one-way clutch to minimize rotational energy loss, the proposed LONG achieves a continuous and stable electrical output from a single mechanical input event. Through systematic parametric analysis, critical design factors, such as the gear ratios, electret potential, and mass of the escapement wheel, influencing the performance of LONG are identified and optimized. In addition, a guide for diode selection in TENG systems is presented by analyzing the effect of electrical rectification circuits based on the dynamic characteristics of diodes on the rectified output. The final optimized system demonstrated enhanced voltage output and extended operation time. The study also verified its feasibility in applications, including lighting large LED arrays and powering low-power electronics. Furthermore, applications utilizing the intrinsic high-voltage output characteristics of TENG systems are experimentally verified by successfully driving a corona discharge-based dust collection system requiring high-voltage input.

The significance of this study lies not only in its functional performance, but also in its design scalability. LONG serves as a prototype for next-generation energy-harvesting systems that require minimal maintenance and can convert unpredictable mechanical stimuli into reliable electrical power. This approach expands the scope of TENG applications to scenarios previously constrained by input intermittency such as wearable devices, infrastructure monitoring, and remote sensing. Future studies should focus on further miniaturization, material integration, and system-level circuit optimization to enhance the overall efficiency and broaden the operational scope of LONG-based systems.

## Supplementary information

Supporting Video 1

Supporting Video 2

Supporting information
