# Supplementary material for: Triboelectric horology: escapement-inspired design strategy for prolonged energy harvesting under irregular mechanical inputs
Source: Microsyst Nanoeng. 2026 Apr 13;12:131. doi: 10.1038/s41378-026-01259-4 (PMC13076662; doi:10.1038/s41378-026-01259-4)
Supplement: Supplementary file 3 — Supporting information [file 41378_2026_1259_MOESM3_ESM.docx]

**Triboelectric Horology: Escapement-Inspired Design Strategy for Prolonged Energy Harvesting under Irregular Mechanical Inputs**

Donghan Lee^1*^, Sanghu Ju^1*^, Dong Yong Park^2*^ Seokhoon Kwon^1^, Yong Woo Jeong^1^, Minsik Choi^1^, Sangjun Lee^1^, Yu-seop Kim^1^, Sumin Cho^1^, Dongik Kam^1^, Zong-Hong Lin^1,3**^, and Dongwhi Choi^1**^

^1^Department of Mechanical Engineering (Integrated Engineering Program), Kyung Hee University, 1732 Deogyeong-daero, Yongin, Gyeonggi, 17104, Republic of Korea

^2^Advanced Mobility Components Group, Korea Institute of Industrial Technology, 320 Techno sunhwan-ro, Yuga-eup, Dalsung-gun, Republic of Korea

^3^Department of Biomedical Engineering, National Taiwan University, Taipei, 106319, Taiwan

^*^D. Lee, and S. Ju contributed equally to this work

^**^To whom all correspondence should be addressed.

Email: [zhlin@ntu.edu.tw](mailto:zhlin@ntu.edu.tw) (Prof. ZH. Lin)

Email: [dongwhi.choi@khu.ac.kr](mailto:dongwhi.choi@khu.ac.kr) (Prof. D. Choi)

**
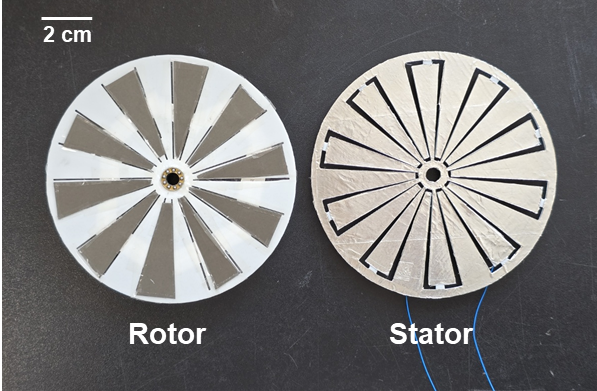
**

**Figure S1.** Photograph of surfaces of the rotor and the stator.


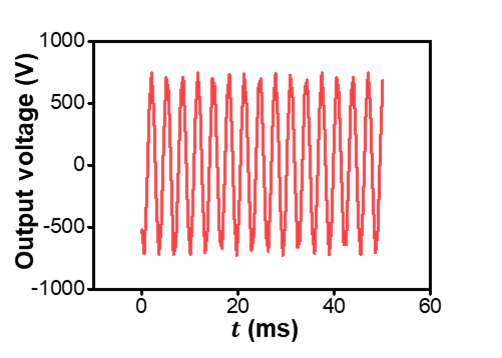


**Figure S2.** Enlarged view of the graph from Figure 1c.


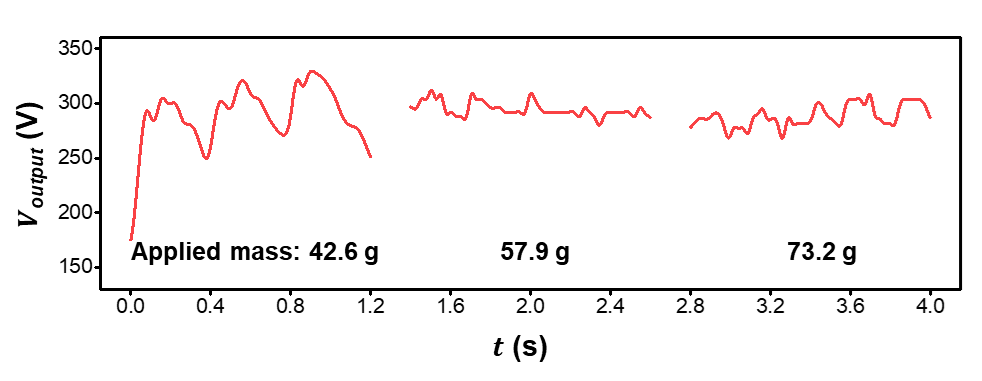


**Figure S3.** Upper envelope of the result shown in Figure 3g.
